# Relationship Between Ovary Size and Anti-Müllerian Hormone Levels in Holstein–Friesian Cows

**DOI:** 10.3389/fvets.2022.828123

**Published:** 2022-06-13

**Authors:** Oky Setyo Widodo, Saeki Nishihara, Dhidhi Pambudi, Ken Takeshi Kusakabe, Yasuho Taura, Yasunobu Nishi, Osamu Yamato, Masayasu Taniguchi, Mitsuhiro Takagi

**Affiliations:** ^1^Joint Graduate School of Veterinary Sciences, Yamaguchi University, Yamaguchi, Japan; ^2^Department of Animal Husbandry, Faculty of Veterinary Medicine, Universitas Airlangga, Surabaya, Indonesia; ^3^Laboratory of Theriogenology, Joint Faculty of Veterinary Medicine, Yamaguchi University, Yamaguchi, Japan; ^4^Department of Mathematics Education, Faculty of Teacher Training and Education, Sebelas Maret University, Surakarta, Indonesia; ^5^Laboratory of Veterinary Anatomy, Joint Faculty of Veterinary Medicine, Yamaguchi University, Yamaguchi, Japan; ^6^Department of Clinical Veterinary Science, Joint Faculty of Veterinary Medicine, Yamaguchi University, Yamaguchi, Japan; ^7^Joint Faculty of Veterinary Medicine, Kagoshima University, Kagoshima, Japan

**Keywords:** ovary size, anti-Müllerian hormone, antral follicle, Holstein-Friesian, ultrasonography

## Abstract

The aim of this study was to verify the association between ovarian size and blood AMH levels in HF cows. Sixty multiparous HF cows from three herds were included in this study. The data required for calculating the ovarian volume included the “major axis (length),” “minor axis (width),” and “thickness” of the ovary. All ultrasonography (US) images were acquired at the outermost ends/poles of both the ovaries and of the follicles (>8 mm) and corpus luteum (CL); concomitantly, the blood was sampled from the jugular or coccygeal vein. Based on the ovarian images of each cow, the following ovarian size patterns were calculated using an image analysis software: (1) total area of both the left and right ovaries, (2) individual size of the large ovary, and (3) individual size of the small ovary. For each ovary area pattern, two properties were assessed: (A) presence of follicles (>8 mm) and CL, which may not secret AMH, in the ovaries and (B) absence of follicles (>8 mm) and CL in the ovaries. Serum AMH levels were measured using enzyme-linked immunosorbent assay. The correlation between ovary size and serum AMH levels was measured in terms of the aforementioned patterns and was evaluated statistically. The results of our preliminary study with ovaries from slaughter-house cows (*n* = 22) revealed that the “thickness” of the ovary was not necessary for estimating ovarian volume and that length and width were sufficient. A strong correlation was observed among ovarian length, width, and thickness (r > 0.96). No significant difference was observed (*p* > 0.05) in the mean ages or parities among the three herds. Among the ovary sizes measured in this study, the highest correlation was found between the total size of an individual large ovary (including follicular and luteal size) and AMH levels (r = 0.387, *p* = 0.002). This is the first study to demonstrate the correlation between total size of individual large ovaries and serum AMH levels in HF cows. US observations of the ovaries will allow for estimation of differences in AMH levels and help predict ovarian activity and superovulation performance of cows.

## Introduction

The profitability of the dairy and beef industries is highly correlated with meat and milk yield, genetic selection, and reproductive efficiency. Optimal reproductive efficiency significantly influences both dairy and beef industries, and it is an essential factor for the management of cow-producing farms. Therefore, successful cow breeding is considered increasingly important not only for traditional breeding programs but also for multiple ovulation embryo transfer (MOET) and/or ovum pick-up and *in vitro* production (OPU-IVP) (1). To achieve these goals, reliable biomarkers exhibiting high reproducibility and heritability associated with the reproductive performance of cows are essential (2, 3).

Anti-Müllerian hormone (AMH) is a 140 kDa glycoprotein belonging to the transforming growth factor-beta superfamily. It is secreted by the ovarian granulosa cells primarily from the preantral and early antral follicles (4, 5). AMH is an endocrine marker that is closely associated with gonadotropin-responsive ovarian reserves and pool size of the growing preantral and small antral follicles (5). Additionally, recent studies have shown that blood AMH levels vary among individual cows, and that they are reliable endocrine markers for the number of ovulation events and embryos produced in both MOET and/or OPU-IVP programs (1, 6–8). Ultrasonography (US) findings indicate that a high antral follicle count (AFC) may be associated with greater reproductive performance, such as higher fertility, shorter open period, and higher responsiveness to superovulation treatment in cows (9–11). In this regard, an increase in the number of 3–8-mm-sized antral follicles is expected to reflect the size of the ovaries. Previous studies have indicated a definite association among serum AMH levels, AFC, the size of the ovaries (total area of the ovaries on both sides), and the size of the ovarian reserve in individual dairy heifers (7, 10, 12–14).

However, simple and portable US units are becoming commercially available to every veterinarian/technician, to improve the degree of image resolution, at a reduced price to further aid current practices in cow farming. Therefore, by monitoring and comparing the AFC and ovarian size of the herd using US, it may be possible to estimate the difference in AMH levels in each cow and determine whether the levels are high or low within the herding group. However, to the best of our knowledge, studies on the association between ovarian size and blood AMH levels, especially those focusing on the presence or absence of follicles larger than 8 mm and/or corpus luteum (CL), the total size of the left and right ovaries, or the individual sizes of the left and right ovaries, have not been conducted to date. We hypothesized that serum AMH levels and total ovarian volume of paired ovaries, excluding luteal volume and the volumes of follicles with a size of 8 mm or larger that did not secrete AMH, may be associated. If an association is observed between the ovarian size measured using US and blood AMH levels, AMH levels can potentially become a simple, inexpensive, and reliable new biomarker of fertility in cows. In addition, levels of serum amyloid A (SAA), one of the most reliable acute-phase proteins (APPs) produced primarily by the liver and other tissues that may affect the AMH levels, and the albumin to globulin (A/G) ratio were determined to describe the inflammatory state of the dairy cow samples. A recent study by our group reported that both an increase in SAA levels and decrease in the A/G ratio during the peripartum period affect AMH levels in dairy cows (15).

Thus, the aim of this study was to determine the association between ovarian size and AMH levels in Holstein–Friesian (HF) cows that (i) would provide veterinary practitioners with more data, enabling better diagnoses, and enhance the production and reproductive performance of dairy cows and (ii) would report the importance of new experiments to unravel the association between ovarian size and AMH levels.

## Materials and Methods

All experiments were conducted according to the regulations of the protection of experimental animals and the guidelines of Yamaguchi University, Japan (No. 40, 1995; approval date: March 27, 2017), and informed consent was obtained from all the farmers.

### Preliminary Study With Ovaries Collected From a Slaughterhouse

Twenty-two bovine ovaries were obtained from a slaughterhouse to produce *in vitro* fertilized embryos. The length, width, and thickness of each ovary were measured using digital sliding calipers in the laboratory. Ovarian measurement data obtained before antral follicular aspiration (3–8 mm in diameter) were statistically analyzed.

### Animals and Management

The study was conducted using 60 randomly selected HF cows that had undergone clinically normal calving from October 2020 to September 2021; they were derived from three commercial dairy herds in Yamaguchi Prefecture, Japan. During the study, the lowest and highest ambient temperatures (°C) ranged from −1 to 9°C in January and 23 to 32°C in August, respectively. The three lactating herds consisted of 22, 22, and 16 cows each. The herds were non-seasonal and were milked twice daily, and the average milk production varied among the three herds by >8,000 kg/cow/year. Throughout the experimental period, the cows were fed a diet of total mixed ration that mainly consisted of grass, whole-crop silage as roughage, and concentrate for dry or dairy cows. The cows were fed *ad libitum* in accordance with the Japanese feeding standard for dairy cows (Japan livestock Industry Association, Tokyo, 2017, in Japanese) to meet their maintenance, growth, and lactation requirements. Two herds were managed in a tie stall with rubber mattresses, and one herd was managed in a free stall. Estrous phase detection was based on hyperemia and swelling of the vulva, mucus discharge, bellowing, and restlessness in the tie stalled herds and standing estrous behavior in the free stalled herd. The cows were inseminated 8–14 h after the appearance of these signs by local artificial insemination technicians. The voluntary waiting period in each herd was set at 60 days postpartum. Monthly follow-ups were conducted for reproductive examination, including treatment of reproductive disorders and pregnancy diagnosis.

### Transrectal Ultrasonographic Evaluation of the Ovaries and Blood Sampling

Per rectum ovarian US evaluations were performed by three technicians using Tringa linear VET^®^ (ESOTE-Pie Medical, Italy) and CTS-800^®^ (Shantou Institute of Ultrasonic Instruments Co., Ltd., China) with a linear array transrectal probe (7.5 MHz transducer); the system was equipped with a USB port for saving the US images; concomitantly, blood sampling was performed from the jugular or coccygeal vein. During US evaluation, areas corresponding to the outermost ends/poles of both the ovaries were examined. Additionally, all US images of follicles >8 mm in diameter and the corresponding CL were recorded for each ovary. In the present US evaluation, we only measured the image area of both sides of the ovary because the results of our preliminary study with slaughterhouse ovaries (*n* = 22) showed that determining ovarian “thickness” was not necessary for estimating ovarian volume and that length and width were sufficient. After the US images were obtained, the ovary was further evaluated for the presence of any large follicles (>8 mm) or CL that were not detected in the initial US assessment. Subsequently, the US images from each cow were analyzed using “WinROOF 2018,” an image analysis and measurement software, to determine and calculate the estimated ovarian area. Based on the images of the ovaries obtained from each cow, the following ovary area patterns were calculated:

(i) total area of both the left and right ovaries
(ii) individual size of the large ovary
(iii) individual size of the small ovary

Area pattern of each ovary was assessed based on two criteria, namely:

(A) presence of follicles (>8 mm) and CL in the ovaries
(B) absence of follicles (>8 mm) and CL in the ovaries

The following formula: (3.1416 × [length/2] × [height/2]) was used to calculate the ovary area (7). This calculation aimed to include or exclude the influence of follicles and CL and obtain a value that more accurately represented the ovarian tissue area. The blood samples were immediately placed in ice for cooling and were transported to a laboratory. After centrifugation at 500 × g for 15 min at 20–25°C, the serum samples were frozen at −30°C until further analysis.

### Measurements of Blood AMH and SAA Levels, and the A/G Ratio

**Figure 1:**
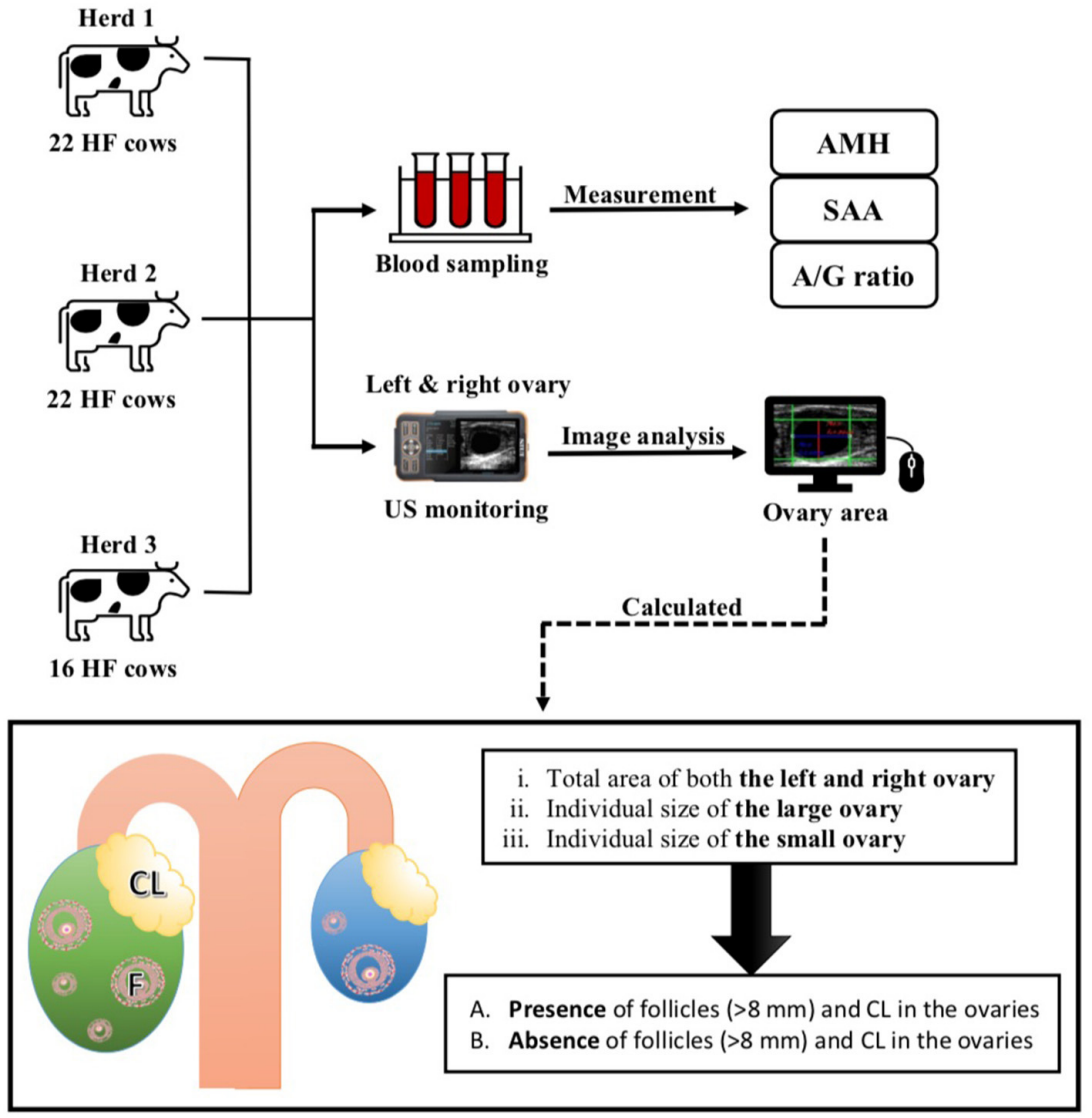
Experimental protocol of the present study. US, Ultrasound monitoring; HF, Holstein–Friesian; AMH, anti-Müllerian hormone; US, Ultrasonography; SAA, Serum amyloid A.

As previously described, the AMH levels were measured using a bovine AMH enzyme-linked immunosorbent assay kit (Ansh Labs, Webster, TX, USA) (1). Undiluted serum (50 μL) was used for the assay, according to the manufacturer's instructions, and the kit had a detection limit of 11 pg/mL and a coefficient of variation of 2.9%. SAA levels were measured using an automated biochemical analyzer (Pentra C200; HORIBA ABX SAS, Montpellier, France) with a special SAA reagent for animal serum or plasma (VET-SAA “Eiken” reagent; Eiken Chemical Co. Ltd., Tokyo, Japan). SAA levels were extrapolated using a standard curve generated using a calibrator (VET-SAA calibrator set; Eiken Chemical Co. Ltd.). Additionally, the A/G ratio was measured using the Labospect 7180 autoanalyzer (Hitachi, Tokyo, Japan) according to the method described in a previous report (15). A summary of the experimental design is presented in Figure 1.

### Statistical Analysis

In the preliminary study, we analyzed the dimensional measurements of the ovaries. The Shapiro–Wilk normality test was conducted for analyzing the length, width, and thickness of slaughterhouse ovaries before the correlation and regression tests. In the correlation analysis, if there was a linear association between these variables, then the test was followed by regression analysis. Three regression models were used to estimate the thickness by determining the length, width, and the combination of length and width. If the thickness could be estimated by the length and width, then the thickness obtained in the next step was omitted.

The preliminary study was followed by statistical analysis to investigate the relationship between ovary size and blood AMH levels. In the present study, the data were first tested for normality (Shapiro–Wilk test), and transformation was performed to obtain normally distributed data. Subsequently, the data were assessed for skewness (using histograms) and normal curve and outlier suspects (using boxplots). Moreover, the suspects were analyzed using the Rosner test. Square root transformation was used for all the non-normally distributed data because they exhibited positive skewness. Transformed data were used in correlation analysis using Pearson's correlation. Regression analysis with the linear model was performed, with “large area” as the dependent variable and AMH level as the independent variable. One-way analysis of variance (ANOVA) was performed to understand the association between ovary size and blood AMH levels. The data of large ovary size were classified into three groups based on original data (non-transformed data). To obtain an even number of samples in each group, the following rule was used:

Group 1, small = X ≤ (mean – 0.44 × SD);
Group 2, medium = (mean – 0.44 × SD) < X < (mean + 0.44 × SD); and
Group 3, large = X ≥ (mean + 0.44 × SD).

The Levene test was used to test the homogeneity of the data. Pair-wise comparisons between groups (small, medium, and large) were analyzed using Tukey's honest significant difference test. Additionally, we analyzed the effect of SAA and the A/G ratio on AMH levels using one-way ANOVA. All statistical data analyzed in this study were coded using R language and were run on R version 4.1.1 for Windows 10. *p*-values <0.05 indicated statistically significant levels.

## Results

### Preliminary Study With Ovaries Collected From the Slaughterhouse

The preliminary study with 22 ovaries collected from the slaughterhouse showed normally distributed in the normality test (*p*-value > 0.05 for all variables; length, *p* = 0.557; width, *p* = 0.322; thickness, *p* = 0.479). Pearson's correlation analysis revealed strong positive association among the length, width, and thickness of the ovaries (r > 0.96), as shown in Table 1. A review of three regression models was performed to estimate the ovarian thickness by determining the length, width, and the combination of length and width, as shown in Table 2. The results indicated that Model 3 was the best regression review model, as it had the highest adjusted *R*^2^. However, Model 1 was simpler and provided an adjusted *R*^2^ slightly lower than that of Model 3. Disregarding the thickness made this study simpler without significantly impacting accuracy, as the thickness could be estimated using the length and width. As a direct impact, the ovary volume can be estimated by only using its length and width. The preliminary observation of ovaries from the slaughterhouse led us to measure the ovarian area in a two-dimensional (2D) context, which comprised the length and width of the largest ovary as recorded using US.

**Table 1:** Pearson's correlation coefficients (r) for length, width, and thickness, with significantly strong positive correlation for 22 examined ovaries collected from a slaughterhouse.

**Parameter**	**Length**	**Width**	**Thickness**
Length	1	0.969	0.982
Width	0.969	1	0.965
Thickness	0.982	0.965	1

**Table 2:** A review of three regression models used to estimate thickness by determining the length, width, and combination of length and width (adjusted *R*^2^).

**Model**	***Adjusted R*** **^2^**
Model 1: Estimate thickness by length	0.961
Model 2: Estimate thickness by width	0.927
Model 3: Estimate thickness by length and width	0.963

### Association Between Ovarian Size and AMH Levels

**Figure 2:**
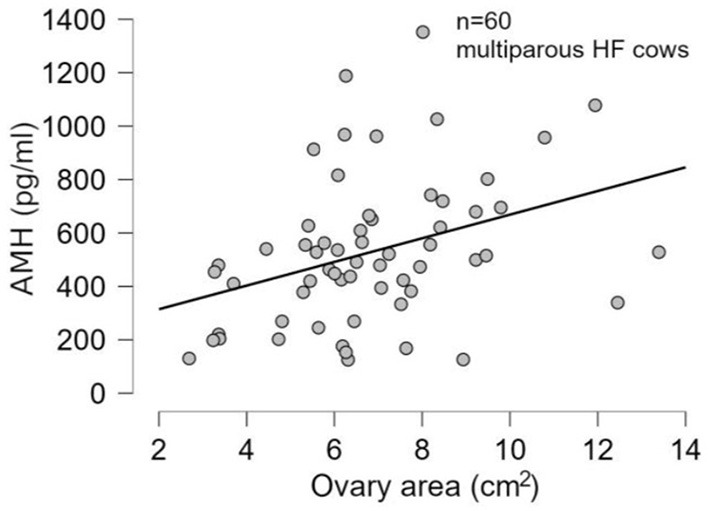
Data indicating a significant correlation (*p*-value = 0.00475) between large ovary size (large area) and blood AMH levels. HF, Holstein–Friesian; AMH, anti-Müllerian hormone.

**Figure 3:**
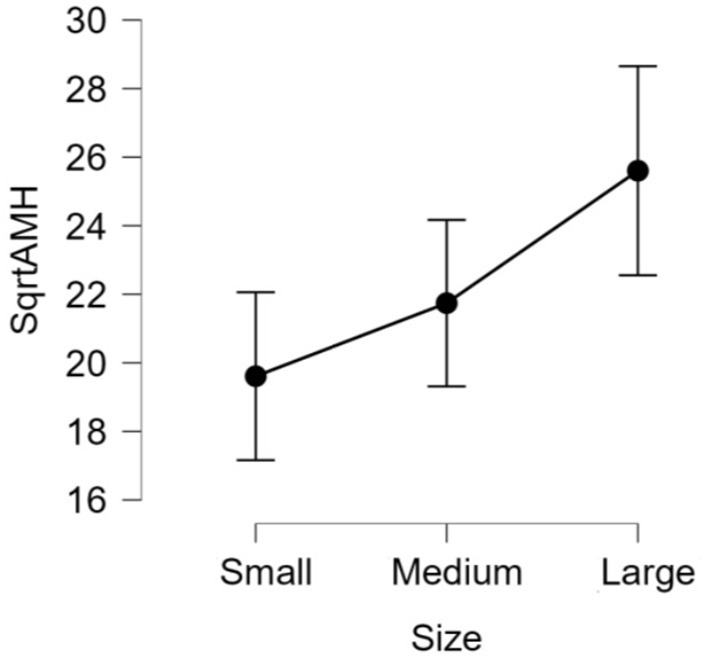
The 95% confidence interval of the mean for each group. The data was transformed into square root of AMH (sqrtAMH) to satisfy the normal distribution assumption. Ovary size: Small = (4.5 ± 1.06), Medium = (6.7 ± 0.6), Large = (9.5 ± 1.6). The differences in all level are significant (*p*-value = 0.00919). AMH, anti-Müllerian hormone.

**Figure 4:**
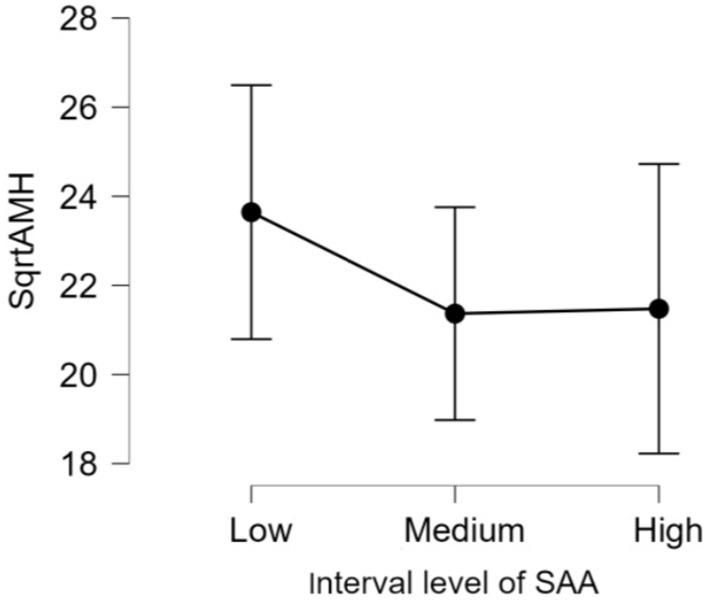
The 95% confidence interval of the mean for each group. The mean and standard deviation (mean ± SD) score of interval level of fourth root of SAA levels are as follows: Low = (1.1 ± 0.2), Medium = (1.5 ± 0.1), and High = (1.9 ± 0.3). The differences in all level are not significant (*p*-value = 0.398). AMH, anti-Müllerian hormone; SAA, Serum amyloid A.

**Figure 5:**
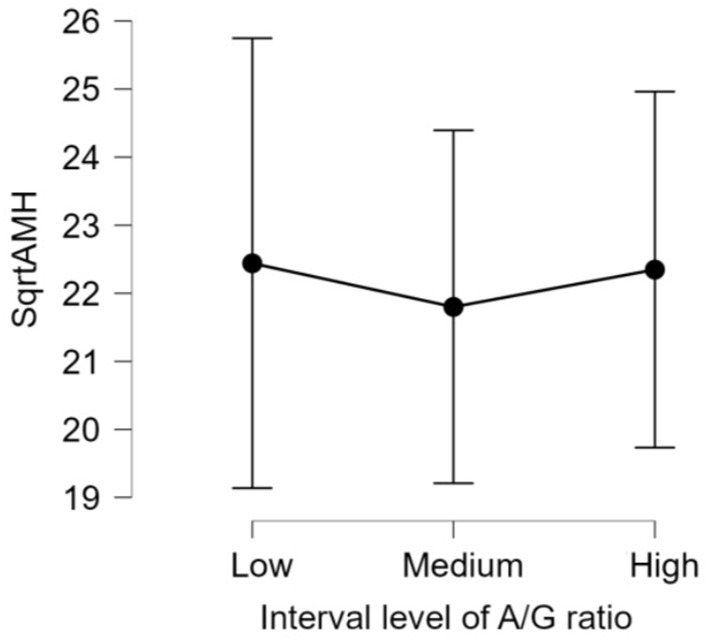
The 95% confidence interval of the mean for each group. The mean and standard deviation (mean ± SD) score of intervals of albumin/globulin (A/G) ratios are as follows: Low = (0.87 ± 0.10), Medium = (1.09 ± 0.05), and High = (1.33 ± 0.12). The differences in all level are not significant (*p*-value = 0.938). AMH, anti-Müllerian hormone; A/G, albumin/globulin ratio.

There was no significant difference (*p* > 0.05) in mean ages (Herd 1: 53.5 ± 19.8, Herd 2: 47.0 ± 23.3, Herd 3: 38.6 ± 22.1) and parities (Herd 1: 2.6 ± 1.4, Herd 2: 2.5 ± 1.6, Herd 3: 2.1 ± 1.5) among the three herds. Thus, the data of 60 HF cows from the three herds were collectively evaluated in the present study. The results of the correlation tests for each variable are shown in Table 3. The highest correlation (r = 0.387, *p* = 0.002) was found between the AMH levels and “large ovary” size/large area (including follicle and luteal volume). Significant positive correlations were observed between AMH levels and “large area,” and AMH levels and “total area.” “Large ovary” size and blood AMH levels were also found to be correlated. “Large area” was an excellent estimator for models because it had a significantly small *p*-value (*p* = 0.00223). However, the model fitness was low (adjusted *R*^2^ = 0.1354), suggesting that the model could probably explain 13.54% of the variations within the data. In other words, 86.46% of the variations could not be explained by this model. The significant correlation between large ovary size and blood AMH levels is represented in Figure 2. The results of one-way ANOVA revealed that large ovary size (large area) had a significant effect on serum AMH levels (*p* = 0.00919). Blood AMH levels in the large group were significantly higher than those in the small group. Groupings and mean intervals for each group are shown in Table 4 and Figure 3. In Figure 3, we can clearly observe a positive correlation between the ovary size and blood AMH levels. The mean intervals of SAA concentrations and the A/G ratio with low, medium, and high AMH levels are shown in Table 5 and Figures 4, 5. No significant differences were observed among the interval level of SAA (*p* = 0.398) and A/G ratio (*p* = 0.938) in groups classified according to AMH levels.

**Table 3:** Pearson's correlation (r) for the association between ovary size and blood anti-Müllerian hormone levels and the significance score for each variable (transformed data).

**Variables**	**AMH**	
	**r**	***p***
Large area	0.387	0.002
Large area (–)	0.122	0.355
Small area	0.206	0.114
Small area (–)	0.110	0.403
Total area	0.301	0.020
Total area (–)	0.160	0.222

*AMH, anti-Müllerian hormone; (–), the area of both follicles (>8 mm) and corpus luteum was excluded*.

**Table 4:** Large ovary size in each classified group.

**Size**	**n**	**Mean ±SD**
1 (small)	18	19.6 ± 4.9
2 (medium)	25	21.7 ± 5.9
3 (large)	17	25.6 ± 5.9

*Data are square-root-transformed (Sqrt.) AMH levels*.

*AMH, anti-Müllerian hormone*.

**Table 5:** Interval level of SAA and A/G ratios in classified groups.

**Groups**	**SAA**		**A/G ratio**	
	***n***	**Mean ±SD**	***n***	**Mean ±SD**
1 (low)	21	23.6 ± 6.3	20	22.4 ± 7.1
2 (medium)	21	21.4 ± 5.3	20	21.8 ± 5.5
3 (high)	18	21.5 ± 6.5	20	22.3 ± 5.6

*Data are square-root-transformed (Sqrt.) AMH levels*.

*AMH, anti-Müllerian hormone; SAA, Serum amyloid A; A/G, albumin/globulin ratio*.

## Discussion

In recent livestock production sites, there is a strong demand for the establishment of biomarkers that are cost-effective and provide information that can improve the productivity of livestock herds. Furthermore, biomarkers in livestock production sites (farms or related inspection agencies) should be easily and quickly obtainable. Our study hypothesized that ovarian size increased concomitantly with increasing AFC in bovine ovaries, thus resulting in an association between the ovarian size itself and blood AMH levels. We found a significant correlation between large ovary size and blood AMH levels in HF cows as per the US monitoring conducted in the farms.

Previously, AFC, AMH levels, and ovarian size were identified as possible phenotypic markers or were associated with fertility and reproductive efficacy in cows (12, 16, 17). AFC has been positively associated with ovarian weight, length, and height; ovarian weight is also highly associated with the AFC as measured by US monitoring using post-mortal ovaries (12). Furthermore, several studies have reported that ovarian AFC and blood AMH levels are associated in individual cows. Information about biomarkers, such as AFC or AMH, is now applied in bovine clinical breeding practice, especially in the implementation of both MOET and OPU-IVP programs in beef and dairy cows (1). Previous reports have indicated that heifers with the lowest AMH levels had smaller ovaries (paired ovaries measured as length and height) than those with higher AMH levels. This indicates that AMH levels were highly variable among the heifers and were positively correlated with ovary size (7). Therefore, this study aimed to examine the association between blood AMH levels and ovarian sizes derived from various ovary patterns of each cow using a previously reported US ovarian sizing method (7), which included (i) the size of the large ovaries, (ii) the size of small ovaries, and (iii) the total size of the left and right ovaries, with or without removal of the CL and follicles (>8 mm) in each ovary. In the present study, the highest correlation was obtained between serum AMH levels and large ovary size, without the removal of the CL and follicles (>8 mm). Furthermore, significant correlations were observed between large ovary size and AMH levels in the three herds under different environmental and feeding conditions. Our study is the first to indicate a significant correlation between AMH levels and large ovary size (left or right). AMH is a suitable biomarker for cow fertility, as well as successful MOET and OPU-IVP programs. Moreover, our results strongly suggest that US is suitable for the measurement of ovarian size and allows for the estimation of AMH levels as a phenotypic marker in cows. However, contrary to our original hypothesis, no significant correlation was found between the larger follicles, excluding the CL and follicles >8 mm, and AMH levels. The reason for this result is unknown, but the effect of subtle differences in the analysis of ovaries by the US technicians cannot be ruled out. In the future, this will need to be taken into consideration in a field test with a larger sample size.

Crane and Muirhead (18) reported that three-dimensional (3D) ovarian US measurements can be used to estimate and evaluate ovarian volume in a routine clinical setting. Moreover, these measurements have the potential to rapidly quantify phenotypic variables associated with fertility, such as AMH levels. As significant correlations were observed between the ovarian volume and ovarian area, calculated based on the diameter of the ovary, we examined the association between ovarian volume and AMH levels using the area of the large ovaries as the ovarian size, excluding the thickness of the ovary.

Monniaux et al. (5) demonstrated that although high, long-term, and intra-individually consistent plasma AMH levels have been reported in cows, some variations in AMH levels exist throughout the reproductive life of animals, especially from gestation to the postpartum period. Previous studies have reported that factors such as nutrition (non-esterified fatty acids), hormones (including endocrine -disrupting chemicals), and diseases (e.g., granulosa-theca cell tumor and mastitis) influence the ovarian reserve of small antral follicles, thereby affecting the AMH levels in animals (7, 19–21). Conversely, SAA is an APP whose synthesis is induced via inflammatory cytokines, such as interleukin (IL)-1, IL-6, and tumor necrosis factor-α. Moreover, it is the most prominent APP in cows and is produced in the liver and other tissues (22). We recently evaluated the changes in both AMH levels and A/G ratios during the perinatal period to identify possible regulatory factors of AMH and reported that the inflammation status, possibly reflected by both an increase in SAA levels and decrease in A/G ratio, was associated with a decrease in serum AMH levels during the perinatal period of dairy cows (15). As cows in the perinatal period (at least 1 month before and after calving) were excluded from the present study, inflammation of the birth canal/genital tract during the perinatal period can be ruled out as the possible cause for the increase in SAA levels. Interestingly, referring to the average SAA levels (22.4 mg/dL) and A/G ratio (0.84) of the cows whose AMH levels decreased during the peripartum period (15), a significant correlation was obtained between the maximum ovarian size and AMH levels regardless of the SAA levels and A/G ratios of each cow as described above. Certainly, in the present study, no significant differences in either SAA levels or A/G ratios were observed among the cows of the categorized groups, namely, the large-, middle-, and small-sized ovaries. Therefore, at least in clinically healthy cows, measuring the ovary size as a biomarker may be useful for estimating AMH levels at all stages, except during the peripartum period.

In conclusion, this is the first study to identify a correlation between large ovary size and blood AMH levels in HF cows. US observations of the ovaries will allow for estimation of the difference in AMH levels and help predict the fertility and superovulation performance of cows.

## Data Availability Statement

The raw data supporting the conclusions of this article will be made available by the authors, without undue reservation.

## Ethics Statement

The animal study was reviewed and approved by all experiments were conducted according to the regulations of the protection of experimental animals and the guidelines of Yamaguchi University, Japan (No. 40, 1995; approval date: March 27, 2017), and informed consent was obtained from all the farmers. Written informed consent was obtained from the owners for the participation of their animals in this study.

## Author Contributions

OW, SN, and MTak: conceptualization. KK, OY, and MTak: methodology. OW, SN, YT, YN, MTan, and MTak: sampling. OW and DP: formal analysis. OW, SN, DP, and MTak: investigation and writing—original draft preparation the manuscript. All authors contributed to writing—review and editing the manuscript.

## Funding

This work was partially supported by grants from JSPS KAKENHI (No. JP18K05971, MTak) and Japan Racing Association (R3-no. 18, MTak).

## Conflict of Interest

The authors declare that the research was conducted in the absence of any commercial or financial relationships that could be construed as a potential conflict of interest.

## Publisher's Note

All claims expressed in this article are solely those of the authors and do not necessarily represent those of their affiliated organizations, or those of the publisher, the editors and the reviewers. Any product that may be evaluated in this article, or claim that may be made by its manufacturer, is not guaranteed or endorsed by the publisher.
